# Late Stent Thrombosis in a Patient with Endovascular Aortic Repair for Blunt Thoracic Aortic Injury

**DOI:** 10.1155/2022/5583120

**Published:** 2022-02-14

**Authors:** Michael H. Chiu, Youri Kaitoukov, Amanda Roze des Ordons

**Affiliations:** ^1^Department of Critical Care, Cummings School of Medicine, University of Calgary, AB, Canada; ^2^Department of Radiology, Cummings School of Medicine, University of Calgary, AB, Canada

## Abstract

Blunt thoracic aortic injury (BTAI) is associated with high mortality and morbidity. Thoracic endovascular aortic repair has become the recommended treatment modality given improved short-term results compared to open repair. We present a case of a 19-year-old male who presented with acute paralysis and multiorgan dysfunction from acute TEVAR thrombosis. Systemic thrombolysis, catheter-directed thrombolysis followed by aspiration thrombectomy, and angioplasty were initially successful in restoring perfusion. However, he developed progressive multiorgan failure related to prompt reocclusion within 48 hours. This case is the first to describe thrombolysis and angioplasty as a management strategy for acute TEVAR thrombosis. We also review the literature surrounding this uncommon complication.

## 1. Introduction

Blunt thoracic aortic injury (BTAI) occurs in 1% of motor vehicle collisions with approximately 20% arriving to hospital alive [1, 2]. Endovascular repair has evolved as a guideline recommended modality for treatment BTAI [2, 3]. Compared to open repair, this modality has resulted in decreased morbidity and mortality, with intermediate-term studies suggesting an improved health-related quality of life [1]. Common complications of endografts include endovascular leaks, ischemia, claudication, stent migration, and fracture [1]. Life-threatening thoracic endovascular aortic repair (TEVAR) thrombosis is a rare complication and mainly limited to case reports. We describe a case of late presenting acute TEVAR thrombosis presenting with acute paraplegia.

## 2. Case Report

A 19-year-old male was involved in a high-speed motor vehicle accident resulting in polytrauma. His injuries included diffuse axonal injury, intraventricular hemorrhage requiring temporary ventriculostomy, complex left acetabular fracture, and a C1 neural arch fracture. He suffered a transection of the thoracic aorta at the isthmus with a pseudoaneurysm. This was managed with a thoracic endovascular aortic repair (TEVAR) with a stent graft. He completely recovered from his injuries with no neurological or physical limitations. Following discharge from hospital, he discontinued the prescribed antiplatelet therapy (aspirin 81 mg daily) and was lost to follow-up.

Eight months after discharge he went for a morning run. While running, he started to get a headache and collapsed. The patient maintained consciousness with complete motor and sensory paralysis at the T8 level. In the emergency department, blood pressure was consistent with a hypertensive emergency. He was anuric from the initial presentation with no changes to his neurological exam.

**Figure 1:**
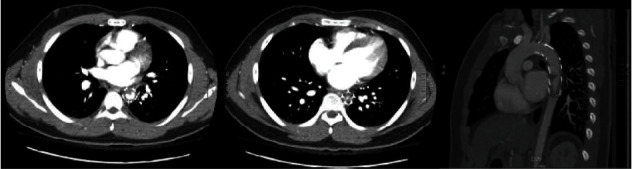
CT scan revealing an endovascular thoracic aortic stent graft spanning the distal arch through the mid descending thoracic aorta. Beginning at the distal third of the stent is circumferential low-attenuation centrally progressing to near complete occlusion at the terminal portion of the stent. Near occlusion at the approximate level of the T7 and T8 intervertebral disc. The superior portion of the thrombus demonstrates a triangular morphology. Small renal infarcts are also noted, seen in the sagittal plane.

CT head and MRI of the spine were unremarkable. CT angiography revealed TEVAR graft thrombosis with near complete occlusion of the thoracic aortic stent graft at the level of T7/T8 (Figure 1). The thrombus appeared to have a triangular/peaked morphology suspicious for fibrin stranding, accompanied by multiple bilateral renal infarcts. Neurology, Cardiothoracic Surgery, and Interventional Radiology were consulted. In view of the imaging findings and the clinical presentation, the running hypothesis was hypoperfusion and acute cord ischemia. Given the absence of cord changes on the MRI, the precise level of potential vascular compromise to the cord was unknown. Lack of cord changes on MRI is presumed to be due to prompt imaging before ischemic injury was detected.

An acute ischemic stroke protocol with systemic alteplase (loading dose 0.09 mg/kg followed by 0.81 mg/kg infusion over 1 hour) was initiated 4 hours after the reported collapse. The patient underwent intubation and proceeded to the catheterization lab for a selective catheterization of the Adamkiewicz artery. Selective angiography revealed no visible spasm or occlusion. 10 mg of alteplase was delivered selectively via the catheter.

The case was discussed with cardiothoracic and vascular surgery for a potential open repair; however, this would require a transfer to a hospital with vascular surgery capabilities. Decision-making of TEVAR restenting and stent extension was challenged by the unknown level of cord ischemia and vascular compromise. Procedural delays and risk of impending permanent paraplegia tipped the momentum towards immediate aortic recanalization with angioplasty with a subsequent plan for open repair once the patient had improved stability for transfer.

**Figure 2:**
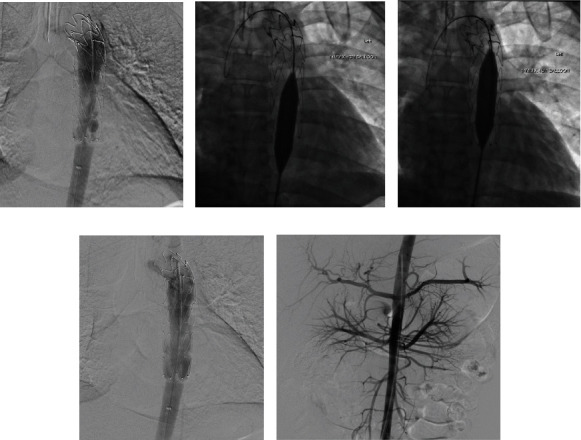
(a) Fluoroscopy post intra-arterial and systemic TPA. (b, c) The stenotic segment in the distal aspect of the stent was treated with 2 overlapping angioplasty deployments using a 14 mm × 4 cm balloon and following of a 16 mm × 4 cm balloon. (d) The final hand-injected aortic run through a 5F straight flush catheter shows mild improvement of the in-stent stenosis. (e) Abdominal aortogram confirming renal parenchymal hypoperfusion in accordance with prior CT findings and complete anuria.

In the interventional radiology suite, the stenotic segment of the aortic graft was crossed with a 5 French KMP catheter over a 0.35 Glidewire. Overlapping angioplasty using 14 mm × 4 cm and 16 mm × 4 cm balloons were performed (Figure 2). Fluoroscopic observation revealed a significant wasting at the initial balloon deployment suggesting a fibrous chronic stricture. Despite residual stenosis on a check-aortogram, no further angioplasty was performed to limit the risk of embolization. Notably, at the survey of the lower limbs in the final stages of angiography, a small partially occlusive embolus was found and aspirated from the right popliteal artery. Following the procedure, the patient was kept on unfractionated heparin, admitted to the ICU, and extubated. Normal consciousness was regained with a nonresolving loss of motor and sensory function from the level T8.

Despite the attempted TEVAR graft reperfusion, the patient developed significant multiorgan failure, including acute renal failure and hyperkalemia. He had refractory hypertension with systolic blood pressures greater than 220 mmHg, requiring esmolol and nitroprusside. He was placed on emergent renal replacement therapy with intermittent hemodialysis, which was changed to continuous renal replacement therapy as he became more hemodynamically unstable.

Sixteen hours after initial presentation, he showed clinical evidence of an acute abdomen with a rising lactate and escalating requirements for vasoactive medications. An emergency exploratory laparotomy revealed copious amounts of free fluid with extensive small bowel ischemia and cecal involvement, attributed to occlusion of the superior mesenteric artery. His distal small bowel was necrotic and resected. The abdomen was partially closed, and the patient returned to ICU.

**Figure 3:**
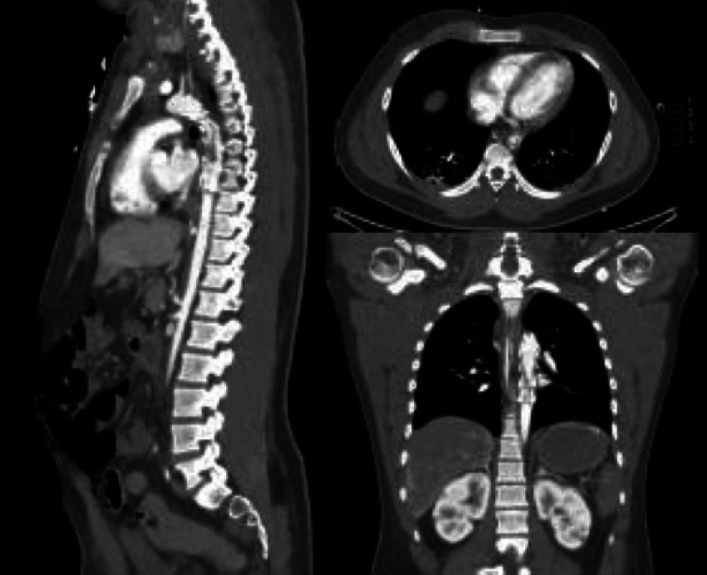
CT scan day 1 postendovascular TPA and angioplasty. An intraluminal filling defect that extend from the proximal descending aorta to the distal tip of the stent graft. There is disappearance of the large bulk of the in-stent thrombus with residual cord-like fibrous components. Abdominal aorta and its major branch vessels are severely diminutive in caliber. New extensive hepatic, renal, and splenic infarcts. Evidence of pneumatosis and hypoenhancement of the bowel.

The patient remained on heparin with the readdition of ASA 80 mg. However, over the course of the next day, his condition continued to worsen with a persistently elevated lactate level and refractory hyperkalemia. A repeat CT scan revealed a large occlusion extending from the proximal descending aorta at the distal tip of the stent graft with disappearance of the large bulk of the in-stent thrombus. There was residual cord-like fibrous components and significantly diminished flow to the major branch vessels, particularly the iliac and lower extremity vessels. New extensive hepatic, splenic, and renal infarcts had progressed, with evidence of diffuse ischemia and several segments of pneumatosis in both the small and large bowel (Figure 3). Discussion between the vascular surgeon, interventional radiologist and critical care team felt that no further intervention would be of benefit. This was discussed with the patient's family, and the patient died shortly after discontinuation of life support.

## 3. Discussion

TEVAR was introduced in 2005 for management of thoracic aneurysmal repair and provides benefits over open repair including decreased operative times, blood loss, and early procedural success. Additionally, TEVAR avoids thoracotomy, cardiopulmonary bypass, and hypothermic circulatory arrest [2]. Early generations of TEVAR stents were intended for aneurysmal degenerative disease instead of traumatic injury. Use of stents in young trauma patients lead to the risk of sizing mismatches with the hyperdynamic nature of patients with healthy vasculature contributing to higher rates of complications [4]. Choosing the appropriate size for the stent can be difficult, as trauma patients are typically hypovolemic and the diameter of the aorta may be up to 30% smaller [3].

Published in 2013, the RESCUE trial used the Medtronic Valiant Captiva stent graft in 50 patients with blunt thoracic aortic injury (BTAI) [5]. Short-term complications were identified in 12% of patients, predominately limited to arm claudication and ischemia [6]. Medium-term outcomes included intramural thrombosis as a rare complication [7]. Long-term results are unknown, especially in the absence of antiplatelet therapy [1]. Furthermore, follow-up and screening has been difficult in the young trauma population. Review of the literature reported 10 prior cases of late TEVAR thrombosis (Table 1) [4, 6, 8–15].

Clinical presentation of stent thrombosis has been variable. Nonocclusive thrombus can be asymptomatic and incidentally found on routine CT [8, 9]. Waxing and waning claudication and intermittent neurological symptoms have also been reported [6, 9–11]. Our patient presented with acute occlusion resulting in motor and sensory paralysis with multiorgan failure, which has also previously been reported in a few cases [4, 11–14] (Table 1). All these cases suffered BTAI from a motor vehicle collision with TEVAR devices from Cook Incorporated, Bolton Medical, Boston Medical, and Medtronic. Time to thrombosis spanned 6 to 39 months with the majority presenting around one-year post stent insertion.

As seen in our patient, a hypertensive emergency can accompany acute TEVAR occlusion. Hypertension post-TEVAR implantation has previously been reported, with the pathophysiology incompletely understood [16]. Post-TEVAR implantation, the dynamic and pulsatile nature of the aorta are lost with stiffening of the aorta leading to adverse remodeling. Physiological implications include increased afterload and myocardial stress [17]. Subacute thrombosis and in-stent stenosis may result in coarctation physiology [6].

Thrombosis was almost exclusively found at the distal portion of the stent [4, 6, 8–12, 15]. Histology from these cases have revealed neointimal formation with fibrosis and evidence of acute thrombus [15]. Researchers have begun to look at the remodeling of the aorta post-TEVAR implantation for BTAI. Early changes are noted at 1.5 years, with lengthening of the aorta, enlarging diameter, and enlargement of distal seal zone [17]. This may result in stent strut and polymer material being exposed, resulting in activation of the extrinsic coagulation pathway. Moreover, mismatch between the prosthetic stent material and the aorta may lead to shear stress activating the intrinsic pathway [18].

The etiology of TEVAR thrombosis is suspected to be due to stent oversizing. Oversizing is typically performed to account for an expanding aorta over time, with the goal of minimizing the risk of pseudocoarctation syndrome. Manufacturers typically recommend a maximum of 20% oversizing. However, oversizing a stent graft can lead to collapse or infolding which has been presumed to be the mechanism of stent thrombosis in a few of the reported cases [4, 6, 8]. Nonadherence to antiplatelet medication is also common in the trauma population, and there has been one reported case of associated coagulopathy from Factor V Leiden mutation [10].

Guidelines including the recently published Society of Vascular Surgery 2021 TEVAR guidelines do not provide a consensus regarding the use of routine antiplatelets for traumatic aortic injury requiring TEVAR [2, 19, 20]. Upon our review of patients (Table 1), low-dose aspirin was the most common antiplatelet of choice for patients post-TEVAR for traumatic aortic injury. The large caliber vessel and the high velocity of blood flow are protective against risk of thrombosis [21]. Prior studies have suggested that chronic anticoagulation may increase the risk of endoleak and hemorrhage increasing the risk of dissection. These concerns also apply to the use of antiplatelets. A recent retrospective study of 388 patients evaluated the safety of patients managed without antiplatelet, low-dose aspirin, and dual antiplatelet therapy postendovascular aortic repair for Type B aortic dissection [21]. No difference in mortality, endoleak, malperfusion, or reintervention was observed between the aspirin and no-antiplatelet group at 12 months. Furthermore, no difference was seen between the dual antiplatelet group compared to low-dose aspirin [21].

Guidelines from the Society for Vascular Surgery recommends follow-up imaging at 1 month and 12 months post-TEVAR implantation with annual imaging thereafter [20]. This imaging interval is helpful in identifying developing complications including obstruction and endoleaks. However, acute complications such as stent thrombosis are difficult to predict and screen for. In addition to medication noncompliance, loss to follow-up is common in the trauma population limiting repeat imaging that can help screen for developing complications.

Most cases of TEVAR occlusion reported in the literature were managed with surgical repair. The literature has reported success with extra-anatomical bypass to expeditiously resolve malperfusion syndromes [12]. Open repair is definitive, whereas a repeat TEVAR may fail. Moreover, endovascular approaches require guidewires, angioplasty, and deployment of stents, which themselves carry the risks of distal thrombus embolization and inability to reestablish perfusion percutaneously [6]. However, repeat endovascular repair has also shown short term success, with three cases reporting prompt restoration of perfusion [4, 9]. Medical management has previously been reported to be unsuccessful in an asymptomatic patient, with a failed 6-month trial of anticoagulation requiring surgical repair [8]. Revascularization of an acute aortic occlusion comes with the risk of ischemia reperfusion injury. Restoration of blood flow may lead to distal embolization, oxidative damage, and inflammation; all of which worsen multiorgan failure.

The case we have reported is the first using a trial of systemic and catheter-directed thrombolysis and angioplasty. Despite initial reperfusion, the immediate course was complicated by reperfusion injury, and shortly thereafter, graft reocclusion and development of multiorgan failure from significant ischemia. Available data has been limited to case reports; however, it suggests that open surgical revascularization may have the best outcome. Angioplasty and catheter-directed thrombolytics may have a role in salvage therapy where emergent surgical or endovascular repair is unavailable. More studies are needed to further study management strategies of these rare complications.

There are continual improvements in endovascular stent technology and many of the reported cases occurred with first-generation TEVAR stents. Improvement in anatomic specificity and improvement in alignment may better mitigate the risk of stent thrombosis.

There is growing evidence to suggest TEVAR thrombosis is an evolving long-term risk in young patients treated for BTAI. Oversizing and nonadherence to antiplatelet therapy are risk factors. More research is needed developing management strategies. This includes determining the ideal interval of surveillance CT imaging to ensure patency and integrity while minimizing cumulative radiation in this young population.

## Figures and Tables

**Table 1:** Case reports of thoracic endovascular aortic repair graft thrombosis for blunt thoracic aortic injury.

Case	Indication for TEVAR	TEVAR and antiplatelet	Time and location of Thrombosis	Presentation	Management	Outcome
Alvarez et al. [6] 17-year-old male Spain	High-speed MVC Aortic rupture distal to the origin of the subclavian artery	Custom 24 mm × 66 mm (oversizing ×30%) TX2 endovascular graft (Cook Incorporated) ASA 100 mg/day	Time: 11 months CT–preocclusive thrombosis of the distal portion of the stent	Abdominal pain and paraparesis over his lower extremities	Anticoagulation and antihypertensive therapy. Right polytetrafluorethylene axillofemoral bypass graft. Open surgical repair with a 20 mm Dacron graft from the ascending aorta to the supraceliac abdominal aorta with removal of the right axillofemoral graft	Asymptomatic 2 years after repair
Marone et al. [8] 32-year-old male Italy	High-speed MVC Posttraumatic 2.5 cm pseudoaneurysm of the aortic isthmus on the lesser curve of the proximal descending thoracic aorta 12 mm distal of the left subclavian artery	24 mm × 104 mm Relay Thoracic stent graft (Bolton Medical Inc.) ASA 100 mg/day	Time: 24 months CT–intragraft atherothrombosis involving the middle and distal third of the aortic stent graft	Asymptomatic and placed on warfarin. Interval progression of size of mural atherothrombosis evolving into multiple intraluminal septa within the aortic stent graft	Failed anticoagulation and converted to open repair with explantation of the aortic stent and reconstruction with a 18 mm Dacron graft	Asymptomatic and well at 3 months
Marino et al. [9] 38-year-old male USA	High-speed MVC Rupture of the aortic isthmus.	Endograft Medtronic Valiant Captivia stent graft VAMF 28 28 C 150 TE ASA 100 mg/day exchanged for warfarin with finding of thrombus	Time: 6 months CT #1 (6 months)—thrombus apposition occupying one third of the graft lumen CT #2 (24 months)—intraluminal thrombus occupied two-thirds of the graft diameter	Asymptomatic. Found on follow-up CT scan	Patient declined open surgery. Redeployment of a conical shape endograft (Medtronic Valiant Captivia stent graft)	Procedure complicated by bilateral distal microembolic lesions at the lower limbs. Treated with low molecular weight heparin infusion. Patient discharged and well at 6 months
Marino et al. [9] 32-year-old male USA	High-speed MVC Traumatic rupture of the aortic isthmus with endograft deployment at the descending thoracic aorta	Relay thoracic stent graft 26 150, (Bolton Medical Inc.) ASA 100 mg/day	Time: 39 months CT–distal device collapse associated with intragraft thrombus apposition	Refractory headache and buttock claudication	Minimally invasive endovascular treatment. A second conical shape endograft Medtronic Valiant Captivia stent graft VAMC 26 22 C 150 TE	Asymptomatic at 10 months with resolved symptoms
Kumpati et al. [10] 14-year-old male USA	High-speed MVC Aortic rupture of the proximal descending thoracic aorta	Two overlapping ilac limb devices (Medtronic Endurant 20 mm × 80 mm proximally and Medtronic AneuRx 20 mm × 57 mm distally) ASA 100 mg/day followed with warfarin for Factor V Leiden	Time: 12 months CT #1 (12 months)—nonocclusive intraluminal thrombus in the distal portion of the endovascular stent graft Time: 24 months CT #2 (24 months)—occlusive thrombus within the distal portion of the stent graft	Scheduled follow-up—asymptomatic with nonocclusive thrombus. Diagnosed with Factor V Leiden and placed on warfarin. Prior ASA 24 months—discontinued anticoagulation and developed claudication	Factor V leiden. Placed on oral anticoagulation. Discontinued by primary care doctor for patient to play competitive sports. Heparin for five days and open repair via a left thoracotomy with explantation of the endovascular stent graft. 20 mm Dacron graft from the distal aortic arch to the mid descending aorta	Stable at 6 months on oral anticoagulation
Reich et al. [11] 24-year-old male USA	High-speed MVC Grade IV traumatic transection of the aortic isthmus	24 mm × 116 mm talent (Medtronic) ASA 81 mg/daily	Time: 14 months CT–obstructive thrombus at the distal end of the stent graft	Collapsed while playing basketball	Open descending thoracic aorta replacement. Removal of the previous stent graft using hypothermic circulatory arrest	Paraplegic at 1 year
Abdoli et al. [12] 29-year-old male USA	Pedestrian vs. MVC Grade 3 blunt injury of the descending thoracic aorta and an intra-aortic thrombus extending caudally	Valiant thoracic stent graft 22 × 100 mm (Medtronic) in the middescending thoracic aorta. Proximal extension using a valiant Thoracic Stent graft 24 × 100 mm ASA 100 mg/day	Time: 9 months CT–near occlusive thrombosis within the distal portion of the thoracic stent	Sudden painful paresthesia below the waist with swelling of the left foot. Subsequent chest pain, renal failure, and GI bleed	Systemic heparin followed by a right axillobifemoral bypass. Long-term warfarin and aspirin. Negative coagulopathy work-up	Brief requirement of renal replacement therapy. Planned explantation of endograft. Completing rehab with improving neurological function
Liesdek et al. [13] 24-year-old-male Netherlands	High-speed MVC Traumatic type B aortic dissection (DeBakey type III)	Not stated	Time: 24 months CT–total occlusion of the thoracic aortic stent graft	Acute complete motoric and sensory loss of both lower limbs while jogging. Development of nausea and vomiting	Emergency surgery to reestablish aortic flow. Left lateral thoracotomy with deep hypothermia. The occluded graft was explanted. Tubular prosthetic graft (24 mm Gelweave, Vascutek Ltd.)	6 months improving neurological status. Patient ambulating with full sensation to his legs
Hostalrich et al. [4] 15-year-old-female France	High-speed MVC Grade 3 blunt thoracic aortic injury just distal to the left subclavian artery	Zenith alpha thoracic stent graft (Cook Incorporated) ASA 100 mg/day	Time: 10 months CT–near occlusive clot at the distal junction between the thoracic stent graft and the native aorta	Thoracic pain and weakness of the lower limbs. Development of multiorgan dysfunction with pulmonary edema with concurrent anuria	Emergent primary stenting with a bare nitinol stent (OPTIMED 22 mm × 60 mm). Treatment with anticoagulation and single antiplatelet	Resolved multiorgan dysfunction. 2 months asymptomatic
Martinelli et al. [14] Martinelli et al. [15] 22-year-old-male Italy	High-speed MVC Blunt thoracic aortic injury below the isthmus	Zenith Cook 22 × 100 mm (Cook Incorporated) endograft ASA 100 mg/day	Time: 6 months CT #1 (6 months)—intimal flap and segmental thrombosis completely occluding the aorta with a valve mechanism CT #2 (8 months)—recurrent partial occlusion of the distal edge of the second graft	Acute ischemic multiorgan failure and complete bilateral lower extremity motor and sensory loss	Emergency endovascular relining of the endograft using another Zenith Cook 22 × 100 mm device with restoration of perfusion except the spinal cord. Open conversion with the endograft explanted. The thoracic aorta was replaced with a 22 mm silver-coated graft	Persistent paraplegia
Chiu et al. 2022 19-year-old-male Canada	High-speed MVC Aortic transection injury at the isthmus with pseudoaneurysm	Unknown	Time: 8 months CT–graft thrombosis with near complete occlusion of the thoracic aortic stent graft	Acute collapse while jogging with complete paraplegia at T8. Multiorgan failure	Emergency systemic and catheter directed tPA followed by aspiration thrombectomy and angioplasty	Death

## Data Availability

All of the figures/tables are included in the main manuscript.
